# Treatment of Epilepsy

**Published:** 1882-06

**Authors:** 


### Selections.

### Treatment of Epilepsy.

Prof. Ball has been investigating the point as to whether the simultaneous action of several drugs is not more efficacious in the treatment of epilepsy than when administered separately, and his results obtained are sufficiently encouraging to deserve attention. The alkaline bromides, particularly those of ammonium and sodium, with belladonna and oxide of zinc, form the basis of treatment. He administers these bromides, of each 10

parts in 300 of water, commencing with teaspoonful doses four times a day, and increasing up to eight or ten doses daily, if the treatment is not followed by improvement within a few days. The belladonna and oxide of zinc are given in pill form, 15 grains of each being made up into forty pills, and of these, two are taken daily, one in the morning, one in the evening; four pills can be given daily in rebellious cases without causing any inconvenience. In congestive cases he employs drastic cathartics, bleeding, or leeches on the temples or behind the ears.

By this treatment he claims to have produced immediate good results, often seen on the second day of treatment. The treatment must not be suddenly discontinued, but the doses should be gradually reduced. For many reasons he prefers this double salt to the other bromides; it does not produce the headache or torpor generally following the prolonged use of the bromide of potassium, and even where a cure is not produced, the double bromide always diminishes the frequency and intensity of the attacks even in cases where the bromide of potassium has failed. The eruption following the use of the potassium salt is rarely seen when the double bromides are used.—*Journ. de Med. de Paris, Jan. 21, 1882.*

